# Successful Operation for the Removal of the Superior Maxillary and Malar Bones

**Published:** 1859-04

**Authors:** Dixi Crosby

**Affiliations:** Professor of Surgery, etc., in Dartmouth College.


### ARTICLE XVII.

*Successful Operation for the Removal of the Superior Maxillary and Malar Bones.* By DIXI CROSBY, M. D., Professor of Surgery, etc., in Dartmouth College.

*Messrs. Editors,*—As the removal of the maxillary and malar bones is one of the infrequent operations in surgery, perhaps the incidents in the following case may prove interesting to some of your readers.

The patient was a Mr. Ira Clifford, of Warren, N. H., a farmer, 55 years of age, and had previously been healthy. There was, however, a scrofulous taint in the family, and during the year 1854 it manifested itself. The cervical glands became enlarged, and the patient fell into a cachectic condition. An appropriate constitutional treatment was resorted to, and in a few months the enlarged glands returned to their original size, and the patient regained his normal tone. Aside from this, no hereditary disease was known to have existed in the family. In the summer of 1855, Mr. Clifford began to experience difficulty in masticating his food on the right side. The teeth, also, upon this side gradually became discolored. In the fall of 1856 he had a tooth extracted, and ever afterward there was a discharge from the right nostril. This consisted of pus, and the greater part of the time was extremely fœtid. In August, 1857, the second molar was extracted, and this operation was followed by a good deal of hemorrhage. In the month of September following, he first consulted a physician, who removed two more of the teeth. In October, an opening was made through the alveolar process into the antrum. Through this opening there was a considerable discharge of pus up to the time of the operation. In November, the cheek began to enlarge towards the inner angle of the eye. Early in December, a profuse discharge occurred from the right nostril, and the canine tooth was drawn. January 26th, 1858, the antrum being much distended, and the cheek continuing to enlarge, an opening was made just beneath the eye, followed by a free discharge of pus. A probe passed in at this opening, made its way through the antrum, and reached the opening previously made through the alveolar. Meanwhile the patient was treated to iodide of potassium, and the antrum injected with a solution of the chloride of zinc.

February 24th, the patient came into the hands of Dr. A. G French, an exceedingly intelligent young physician residing at Warren, who subsequently had charge of the case. To his assiduous care and skill is due much of the success which attended the operation. February 25th, Dr. French enlarged the opening upon the cheek, but advised the patient to have the diseased maxilla removed immediately. I was called in consultation with Drs. French and Stearns, and fully coincided with them as to the propriety of an operation. Some palliative measures were advised; but in a few days the patient became *im*patient, and on the 5th of March the operation was performed. There were present at the operation, Dr. A. G. French, the attending physician; Dr. Peter L. Hoyt and Dr. A. B. Crosby. Messrs. Shaw, Fellows, Smith, Crosby and Whipple, medical students, were also present and assisted.

The patient being firmly secured in a chair facing a window, chloroform was exhibited until complete anæsthesia was induced. An incision was then made, commencing at the external angular process of the frontal bone, and terminating at the angle of the mouth. The incision was in the form of a curve, the convexity being backward. Another incision was now made, commencing at the internal angular process of the frontal bone, passing down the side of the nose and separating the ala, finally splitting the lip in the course of the philtrum. An incision was now made, one inch in length, commencing upon the malar bone and passing backward along the zygoma. The whole cheek was then dissected upward, detaching it from the bone, and turned up over the eye. A branch of the coronary artery, and one of the facial, were divided, but were readily controlled by pressure. The anterior wall of the antrum was found to be partially destroyed, apparently by caries. The orbital wall was in the same condition, and the remaining walls were much expanded. On dissecting up the cheek, several ounces of offensive matter escaped from the antrum, and for a time threatened to deluge the patient's mouth. The eye being gently separated from the external wall of the orbit, a pair of strong bone pliers were applied, so as to divide the articulation between the malar and frontal bones. The same instrument was employed to divide the zygoma. One blade was introduced into the nostril and the other into the orbit, and the intervening bone cut through. The middle incisor tooth of the right side was now extracted, and a very strong pair of the bone pliers cut through the alveolar and palate processes of the bone. The remaining points of attachment to the soft parts were easily separated by the knife, and the diseased mass was removed. From the fact that the orbital was partially broken down, portions of bone remained adherent. These were removed, until all the parts remaining were healthy. The operation occupied seventeen minutes. Dr. French and my son, Dr. A. B. Crosby, took charge of the dressing. Five twisted sutures were employed: two in the lip, one on the side of the nose, and two in the curved incision. This brought the cheeks fully into position over some tow which had been introduced into the cavity. Several interrupted sutures were introduced, so as to coaptate the edges of the wounds throughout their whole extent. A simple water dressing was applied, and the patient removed to his bed. For the subsequent history of the case, I am indebted to Dr. French, who was so kind as to keep a record of it.

"March 5th—Three hours after the operation. Re-action is well established in the flap. The pulse is 90 in the minute, and of good tone. The hemorrhage has been checked by injecting a solution of alum.

"6th, 9 o'clock, A. M.—Patient rested well during the night. His pulse is 85 in a minute. Have given him food and drink through a tube. The wounds have healed throughout their whole extent. Have dispensed with all medicine, simply enjoining quiet. 11 o'clock, P. M.—Pulse 100 in the minute. Have given him a laxative of rhubarb and castile soap. Have removed the tow from the mouth, and the discharge seems healthy.

"7th, 5 o'clock, A. M.—Patient has not rested well during the night. Has a pulse of 60 in the minute; extremities cold, and a disposition to sink. Have resorted to friction, and administered brandy and quinine. 8 o'clock, A. M.—Pulse 70 and of sufficient tone.

"8th, 9 o'clock, A. M.—Patient has slept well. Pulse 70. Have continued the stimulants and tonics.

"9th.—Patient is doing well. Pulse 65, and of good tone. Have removed the middle suture on the cheek. The parotid gland is somewhat enlarged.

"10th.—Patient continues improving. Have removed the remaining sutures, and applied adhesive strips and collodion. The discharge from the cavity is now moderate and healthy. Have given an enema to procure a movement of the bowels. Have ordered porter and quinine.

13th, 15th and 18th.—Every thing has continued favorable, and the patient is constantly improving."

Some time after this, I received a letter from Dr. French, saying that the wound over the zygoma had re-opened. I suggested there might be a spiculum of bone which had caused the mischief, and advised a thorough probing, and its removal, if found to be the cause. I received another letter, bearing date of May 3d, from which I make the following extract:

"Mr. Clifford came down to see me last Saturday, and feels first rate; says he has no doubt but he should be able to walk four or five miles! Has a good appetite; rests well, and is free from any pain. The opening upon his face has closed up."

I have since learned that the patient is quite well, and the deformity much less than might have been expected. Judging from the appearance, I do not think the disease was cancerous in its character. I intended to have examined the morbid specimen under the microscope, but was prevented from doing so until it was spoiled. Considering the progress the disease had made before the operation, the result has been much more favorable than we had any right to expect. [*Boston Med. and Surg. Jour.*

